# Effectiveness of cold atmospheric plasma in decontaminating enterococcus faecalis colonized collagen and PTFE membranes used in guided bone regeneration: a comparative in vitro investigation

**DOI:** 10.1186/s40729-024-00576-5

**Published:** 2024-11-14

**Authors:** Jan-Tobias Weitkamp, Adrian Hogreve, Johannes Spille, Salih Veziroglu, Oral Cenk Aktas, Christian Flörke, Kim Rouven Liedtke, Jörg Wiltfang, Aydin Gülses

**Affiliations:** 1 Clinic for Oral and Maxillofacial Surgery, University Hospital Schleswig-Holstein, Campus Kiel, Kiel, Germany; 2 Chair for Multicomponent Materials, Technical Faculty, Christian-Albrechts-University, Kiel, Germany; 3 Clinic for Orthodontics, University Hospital Schleswig-Holstein, Campus Kiel, Kiel, Germany; 4 Clinic for Orthopedic and Trauma Surgery, University Hospital Schleswig-Holstein, Campus Kiel, Kiel, Germany

**Keywords:** Cold atmospheric plasma, Guided bone regeneration, Bacterial infection

## Abstract

**Purpose:**

Wound healing disorders caused by bacterial infections in dental surgery, especially where membranes are used, are a common issue in oral surgery. Cold atmospheric plasma (CAP) offers a non-invasive solution for surface decontamination, including dental implants. The aim of this study was to evaluate the antibacterial effectiveness of CAP on various clinically applied membranes made of collagen and polytetrafluoroethylene (PTFE).

**Materials and methods:**

To assess the antibacterial properties of CAP, enterococcus faecalis were seeded on different membranes: Memlock (collagen), Memlock Pliable (collagen), Agronaut (collagen), and PermaPro (PTFE); n = 4. After in vitro cultivation for 6 days, CAP using a kINPen^®^ MED with an output of 5 W was applied 5 min and 10 min. Bacterial colony-forming units (CFU) were quantified to detect decontamination effectiveness. In addition, live and dead staining as well as scanning electron microscopy (SEM) of membranes was performed for validation and surface texture analysis.

**Results:**

Bacterial colonization was highest on collagen-based membranes (CFU Memlock: 14.38 ± 8.91). The results showed that CAP significantly reduced bacterial colonization on all membrane types after 10 min application of CAP; Memlock (CFU after 10 min 0.22 ± 0.16^10^6^; *p* = 0.0256), Argonaut (CFU after 10 min 0.02 ± 0.01^10^6^; *p* = 0.0129) and PermaPro (complete bacterial decontamination; *p* = 0.0058). This was paralleled by fluorescence and scanning electron microscopy. CAP was most effective on smooth membrane surfaces as SEM revealed.

**Conclusion:**

CAP thus offers a non-invasive, cost-effective method to reduce bacterial infections in guided bone regeneration using membranes.

**Graphical Abstract:**

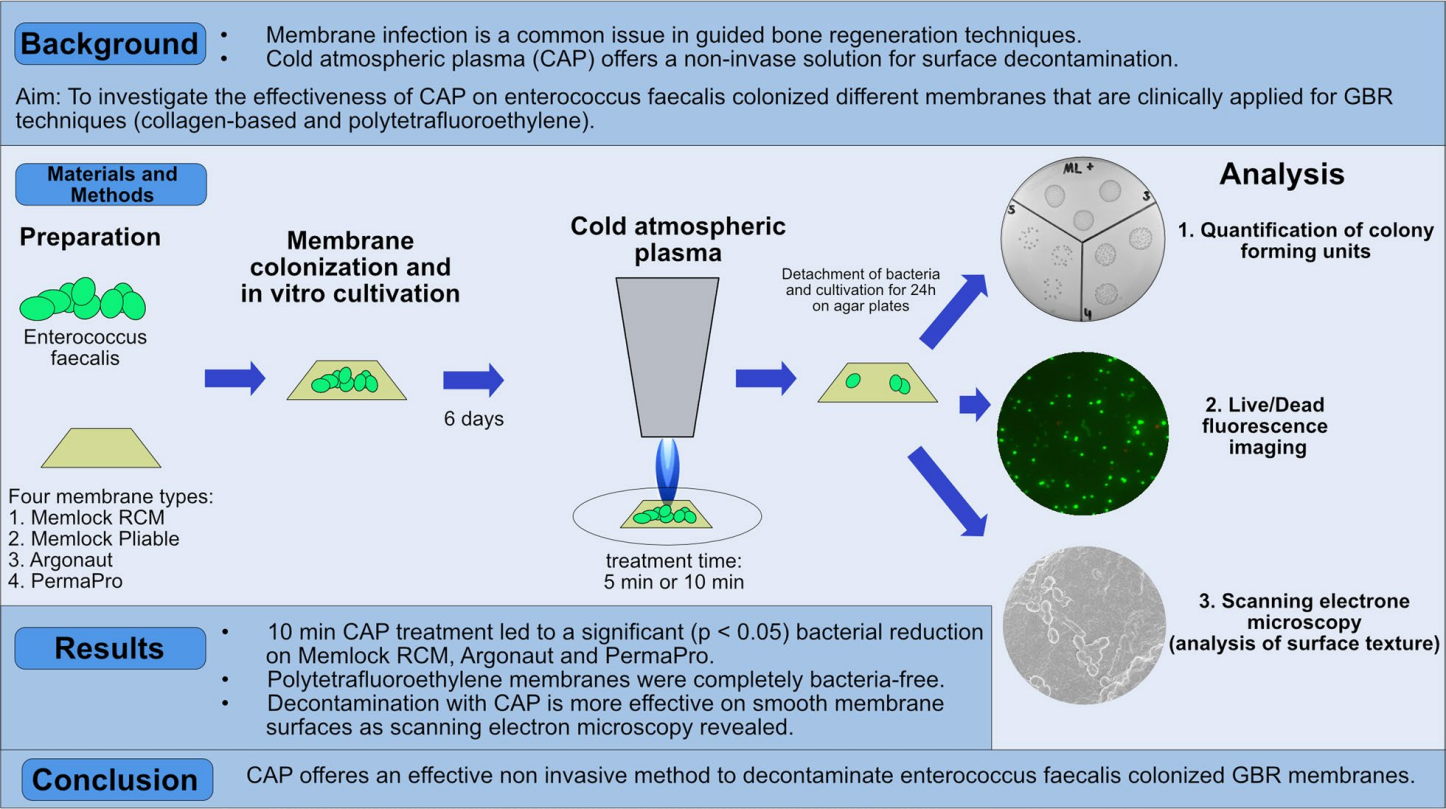

## Introduction

Guided bone regeneration (GBR) is commonly employed in implant and periodontal surgery, where membranes are used to create a barrier between bone and soft tissue. The primary goal of this approach is to prevent the infiltration of soft tissue progenitor cells into the bone defect, allowing new bone formation, which occurs more slowly than adjacent oral mucosa. However, the complex bacterial environment of the oral cavity presents a high risk of membrane colonization or wound infections, which can significantly hinder bone regeneration. Clinical studies emphasize the need for anti-infective therapies in GBR. For example, De Santis et al. reported bacterial colonization of polytetrafluoroethylene (PTFE) membranes in up to 65% of cases 5 weeks postoperatively, leading to reduced attachment gains in periodontal surgery [1]. Similarly, Zuchelli et al. observed bacterial colonization in bioabsorbable membranes, particularly when intraoral exposure occurred [2]. These findings highlight the need for either efficient membrane decontamination or the development of bactericidal membranes. Any decontamination procedures must effectively reduce bacterial load without damaging surrounding tissues.

Cold atmospheric plasma (CAP) is an emerging technology with significant potential in oral surgery, particularly for its antimicrobial and wound-healing properties [3, 4]. CAP generates reactive oxygen and nitrogen species at room temperature, which are bactericidal and virucidal without causing thermal damage to tissues. This makes it ideal for the use in sensitive areas like the oral cavity [5, 6]. In recent years CAP gained attention for the treatment of peri-implantitis of dental implants, a condition where a biofilm induced infection on the implant surface leads to a chronic inflammation resulting in osteoclast activity and bone loss. Numerous in vitro studies reported promising results in reduction of biofilms on titan surfaces [7–10]. In addition, CAP might even synergistically support tissue regeneration by enhancing cell proliferation and extracellular matrix synthesis [11, 12]. Current research is focused on optimizing CAP delivery methods and evaluating long-term clinical outcomes. While early results are promising, more clinical trials are needed to fully establish the efficacy of CAP in peri-implantitis treatment.

Given the promising evidence of CAP in peri-implantitis, its potential application as an anti-infective treatment for GBR membranes is an intriguing prospect. Nowadays a broad spectrum of GBR membranes is clinically applied. They can be divided into natural and synthetic or bioresorbable and non-resorbable. Most frequently, membranes manufactured from natural structural proteins like collagens are used in clinical praxis due to their excellent biocompatibility. Different textures and origins like bovine and porcine offer varying stiffness and degradation. On the other hand, PTFE membranes can be used for larger size defects due to their structural integrity and are suitable for open wound healing where a primary wound closure is not possible. In an in vitro model it was recently shown that CAP led to significant reduction of colony forming units (CFU) on enterococcus faecalis-contaminated type I/III collagen membrane used for GBR [13]. These preliminary findings suggest that CAP treatment could be beneficial in clinical practice. However, there is currently no evidence in the literature regarding the efficacy of CAP in different membrane types or its optimal application time intervals. Therefore, the aim of this study is to:

- comparatively investigate the effectiveness of CAP in decontaminating clinically applied natural and synthetic GBR membranes,
- to determine the most suitable material type by the risk of membrane exposure in terms of management with CAP hypothetically.

## Materials and methods

### Bacterial contamination

Bacterial contamination was performed as previously described [8, 13, 14]. At first four different membranes (1. Argonaut^®^ porcine pericardium collagen membrane; Mem-Lok^®^ RCM porcine collagen membrane; Mem-Lok^®^ Pliable bovine Type I collagen membrane; PermaPro^®^ synthetic non resorbable PTFE membranes; all Biohorizons biologics) that are clinically applied for GBR were cut into 5 × 5 mm pieces. At first, a sterile nutrient solution (10 ml Brain–Heart-Infusion Broth, Carl Rot, Karlsruhe, Germany) along with 100 µl of a bacterial culture of *Enterococcus faecalis* ATCC 29212 (Leibniz Institute DSMZ, Braunschweig, Germany) was incubated at 37 °C for 24 h Then day, a 10 µl sample of the incubated bacterial culture was transferred onto an agar plate (Sarstedt AG & Co. KG, Nümbrecht, Germany) The resulting *E. faecalis* colonies were then prepared for the bacterial contamination. All specimens were cultured with 2.5 ml of BHI broth and 2.5 µl of the *E. faecalis* overnight culture (1:1000 dilution). To ensure optimal bacterial growth, the plates incubated at 37 °C for 6 days in a shaker (Rotamax 120, Heidolph Instruments GmbH & CO. KG, Schwabach, Germany). BHI was changed every 2 days. All experiments were conducted using n = 4 specimen per experimental group. One specimen per experimental group was not colonized with bacteria and served as a negative control.

### Cold atmospheric plasma

**Fig. 1:**
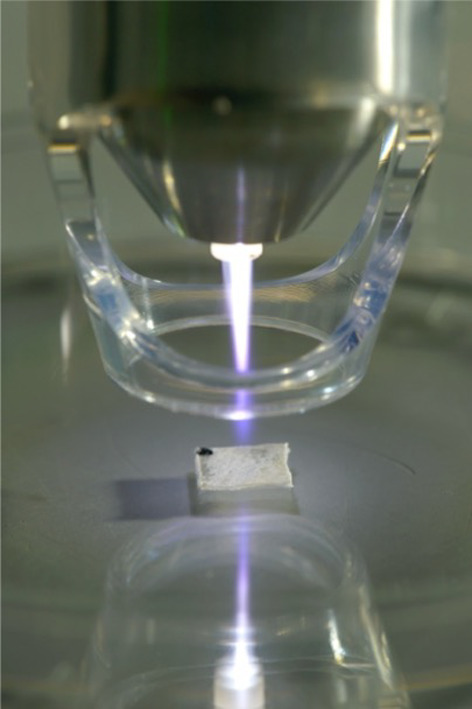
Representation of plasma application using a kINPEN®

After bacterial colonization, specimens were washed using phosphate buffered saline and were then prepared for cold atmospheric plasma treatment. Specimen were placed in 6 well plates and then CAP (argon plasma) treatment was performed using a kINPen^®^ MED (neoplas tools GmbH, Greifswald, Germany). One experimental group (n = 4) received 5 min CAP with 5 W and a second group (n = 4) received 10 min CAP. The time intervals were chosen based on a previously performed study, where a 5 min treatment interval reduced the bacterial load significantly by two third on collagen membranes [13]. A 10 min interval was additionally added in the present study to detect if further decontamination was possible with longer exposure to CAP. The distance from the device to the specimens was 1 cm (Fig. 1). A positive control group (n = 4) per membrane type received no treatment and served as positive control group.

### Colony-forming units

To quantify colony forming units of contaminated membrane types samples were washed with PBS and then placed in an ultrasonic bath (Branson 2210 R-MT Ultrasonic Cleaner, Branson Ultrasonics Corporation, Danbury/CT, USA) for 15 min to ensure bacterial detachment. Afterwards, a three-step dilution series was prepared for each membrane using SafeSeal reaction tubes (2 ml, Sarstedt AG & Co. KG, Nümbrecht, Germany). The first dilution (10⁻^2^) was prepared by adding 10 µl of the bacterial solution to 990 µl of sterile NaCl solution and thoroughly mixing it using a vortex shaker. The second dilution (10⁻^3^) was produced by mixing 100 µl of the first dilution with 900 µl of sterile NaCl solution. Finally, the third dilution (10⁻^4^) was made by mixing 10 µl of the second dilution with 900 µl of NaCl solution. From each dilution, 3 × 25 µl of the respective dilutions were applied to prepared BHI agar plates (Sarstedt AG & Co. KG, Nümbrecht, Germany). These plates were then incubated at 37 °C for 24 h in an incubator. After the incubation period, the colony-forming units were counted using a colony counter (BZG 25, Bender & Hobein AG, Zurich, Switzerland) to determine the number of viable, colony-forming bacteria.

### Live/dead immunostaining and fluorescence imaging

In addition to CFU quantification, Live/Dead staining using a BacLight^™^ Bacterial Viability Kit (L7012) according to manufacture’s instructions. Briefly, Samples were washed in PBS and then placed on microscopy slides. To each 5 µl of dye mixture was applied. Then samples were covered with a cover slip. The prepared slides were then incubated in the dark at room temperature for 10 to 15 min and immediately prepared for fluorescence microscopy.

### Scanning electron microscopy

Bacterial colonization on all different membrane types was semi quantitatively evaluated using scanning electron microscopy (SEM). After incubation in a humified atmosphere of 37 °C at 5% CO_2_, the culture medium was removed and bacteria were fixed with glutaraldehyde (Sigma, St. Louis, USA) 3% in PBS at a pH value of 7.4 for 24 h. After removal of the glutaraldehyde solution, the samples were dehydrated in an ascending alcohol dilution for 300 s for each series. After that, drying with hexamethyldisilane for 1 min (Sigma-Aldrich, St. Louis, USA) and a gold vapor deposition with a thickness of 15 nm (SCD 500, CAL-Tec, Ashford, UK) were performed and SEM analysis (Jeol, Freising, Germany) was conducted at a voltage between 10 and 15 kV.

### Statistics

All experiments were conducted in quadruplicates (n = 4 per experimental group). Power analysis was performed with SPSS (IBM) to determine the smallest sample size that is suitable to detect the effect of a given test at the desired level of significance. All data were tested for normality using Shapiro–Wilk test. Statistical analysis of CFU data was performed using Graph Pad prism 7 program (San Diego, CA, USA). One-way analysis of variance (ANOVA) with Bonferroni’s multiple comparisons was used to compare means among the independent experimental groups. If there was no normal distribution, the Kruskal–Wallis test was used with Dunn’s multiple comparison. Differences were considered significant if *p* ≤ 0.05. Quantitative data in the text are presented as mean and standard deviation (SD).

## Results

**Fig. 2:**
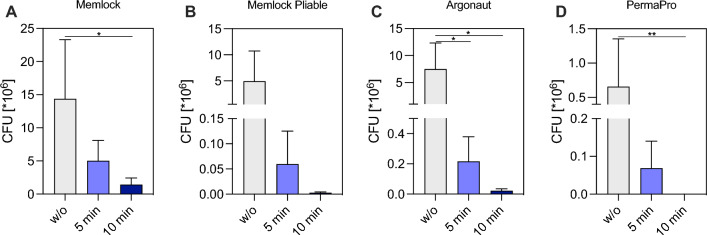
Effect of cold atmospheric plasma on colony forming units of *E. faecalis* on different membranes. Decontamination of the following membranes **A** Memlock (collagen), **B** Memlock Pliable (collagen), **C** Argonaut (collagen) and **D** PermaPro (polytetrafluoroethylene) after administration of 5 min and 10 min CAP compared to a positive control group without (w/o) treatment. Mean + SD; n = 4; Asterisks indicate groups that are statistically significant to one another * = *p* < 0.05, ** = *p* < 0.01; One-way ANOVA (A-C) and D Kruskal–Wallis test

Effectiveness of CAP treatment was detected by CFU of enterococcus faecalis and L/D staining as well as SEM analysis of bacterial seeded membranes. Quantitative analysis of CFU revealed significant differences of the different membrane types (for numerical data please see Table 1). First of all, collagen-based membranes Memlock, Memlock Pliable and Argonaut were greater colonized compared to PermaPro after 6 days of in vitro cultivation (Memlock 14.38 ± 8.912^10^6^ versus PermaPro 0.66 ± 0.69^10^6^; *p* = 0.0368). The application of CAP of bacterial contaminated membranes resulted in a reduction of CFU on all four membrane types (Fig. 2A–D). After 5 min of CAP treatment of colonized Argonaut CFU were already significantly lower compared to the untreated control group (Argonaut w/o CAP 7.5 ± 4.83^10^6^ versus Argonaut treated with CAP for 5 min 0.22 ± 0.16^10^6^; *p* = 0.015; Fig. 2C). After 10 min of CAP treatment CFU were significantly reduced on Memlock (Memlock w/o CAP 1.42 ± 1.0^10^6^ versus Memlock treated with CAP for 10 min 0.22 ± 0.16^10^6^; *p* = 0.0256) and Argonaut (Argonaut w/o CAP 7.5 ± 4.8^10^6^ versus Argonaut treated with CAP for 10 min 0.02 ± 0.01^10^6^; *p* = 0.0129) On the PTFE membrane were zero bacteria detectable (*p* = 0.0058) after 10 min of CAP treatment compared to the untreated experimental group PermaPro (PermaPro w/o CAP 0.66 ± 0.69^10^6^; Fig. 2D). Decontamination of Memlock Pliable also showed a tendency of CFU reduction after 5 and 10 min (Memlock Pliable w/o CAP 4.96 ± 5.8^10^6^ versus Memlock Pliable treated with CAP for 10 min 0.003 ± 0.002^10^6^) without reaching statistical significance (Fig. 2B).

**Table 1:** Colony forming units quantification of untreated and treated membranes

Sample ID	CFU *10^6^ w/o treatment	CFU *10^6^ + 5 min CAP	CFU *10^6^ + 10 min CAP
Memlock			
#1	20.27	6.75	1.91
#2	6.81	3.92	1.25
#3	6.71	1.29	0.11
#4	23.73	8.19	2.43
Mean ± SD	14.38 ± 8.91	5.04 ± 3.06	1.42 ± 1.0
Memlock pliable			
#1	11.77	0.1	0.005
#2	7.7	0.13	0.003
#3	0.05	0.003	0.002
#4	0.18	0.005	0.002
Mean ± SD	4.93 ± 5.79	0.06 ± 0.06	0.003 ± 0.002
Argonaut			
#1	12.93	0.28	0.01
#2	3.37	0.42	0.04
#3	10.21	0.08	0.01
#4	3.48	0.09	0.03
Mean ± SD	7.5 ± 4.83	0.22 ± 0.16	0.022 ± 0.013
PermaPro			
#1	1.59	0.17	0
#2	0.18	0.06	0
#3	0.79	0.02	0
#4	0.07	0.02	0
Mean ± SD	0.66 ± 0.69	0.069 ± 0.072	0

**Fig. 3:**
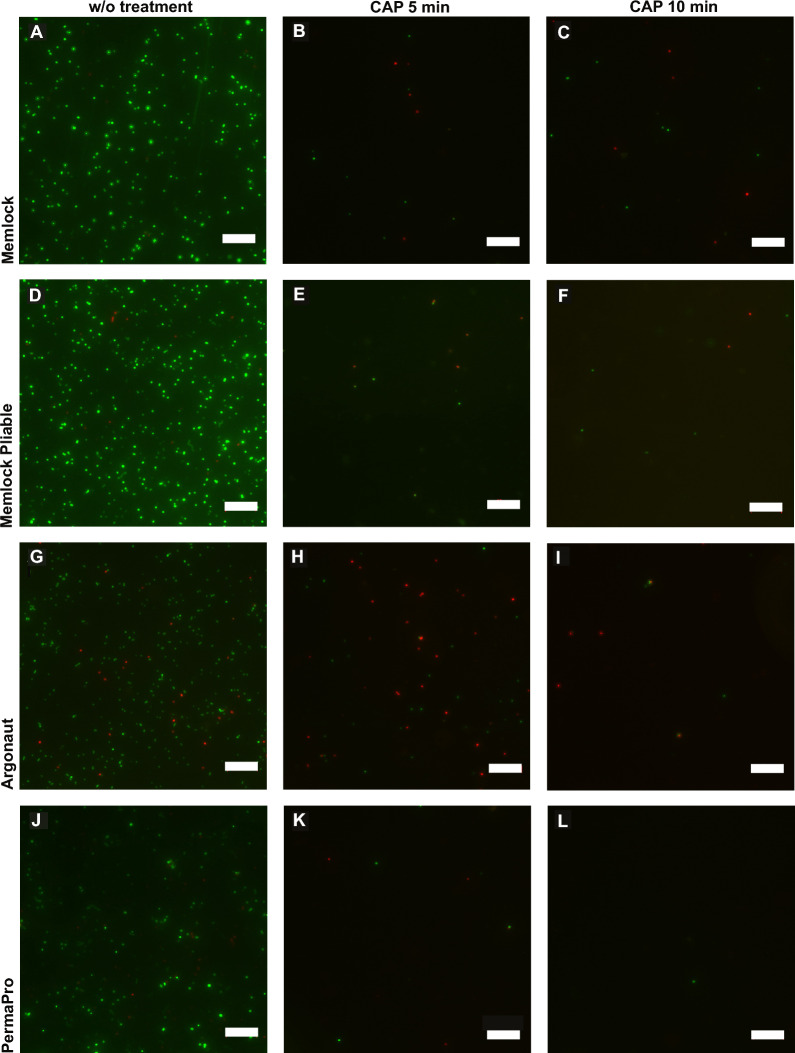
Live/Dead staining of bacteria colonized membranes. Treatment with cold atmospheric plasma revealed a reduction in viable bacteria (green) and an increase in dead enterococcus faecalis (red). Without treatment (A, D, G, J). Images after 5 min application of cold atmospheric plasma (B, E, H, K). Images after 10 min application of cold atmospheric plasma (C, F, I, L). Representative images. w/o = without treatment, CAP = cold atmospheric plasma. Bar 2 µm

**Fig. 4:**
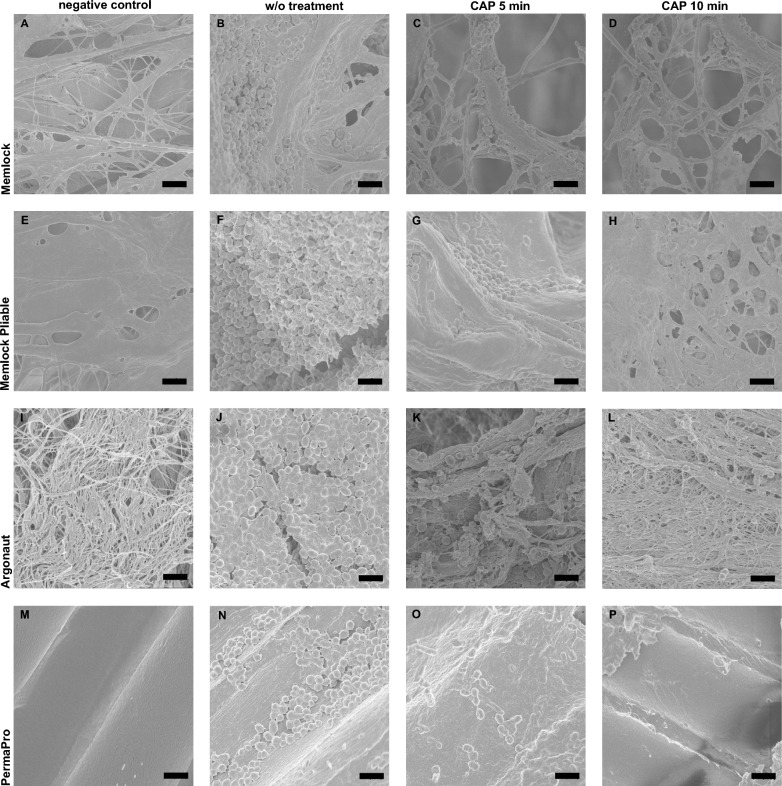
Representative images of scanning electron microscopy of bacteria colonized membranes. Negative control groups without bacterial colonization (A, E, I, M). PTFE (M) membrane displays a smooth surface compared to specimen made of collagen (A, E, I). Treatment with cold atmospheric plasma revealed a reduction of enterococcus faecalis. Without treatment (B, F, J, N). Images after 5 min application of cold atmospheric plasma (C, G, K, O). Images after 10 min application of cold atmospheric plasma (D, H, L, P). Representative images. w/o = without treatment, CAP = cold atmospheric plasma.w/o = without treatment, CAP = cold atmospheric plasma. Bar 1 µm

Decontamination was paralleled by L/D staining and SEM analysis. L/D staining of Memlock and Memlock Pliable showed great bacterial colonization (Fig. 3A, D). CAP treatment led to a reduction in viable bacteria in all four different specimen (Fig. 2). As CFU quantification revealed no viable bacteria were observed on PermoPro after 10 min of CAP treatment. Similar results were observed in SEM analysis (Fig. 4). Surface evaluation revealed a smooth surface of PTFE membranes, while membranes made of collagen displayed an ideal rough surface for bacterial colonization.

In summary, CAP treatment of 10 min resulted in a significant decontamination of all four different membrane types.

## Discussion

This study aimed to investigate the effectiveness of Cold Atmospheric Plasma (CAP) as a decontamination method for different types of membranes used in guided bone regeneration (GBR). The results confirm that CAP treatment significantly reduces bacterial colonization on different GBR membranes, including collagen-based membranes like Memlock, Memlock Pliable, and Argonaut, as well as synthetic PTFE-based membranes like PermaPro using an established in vitro model.

The quantitative analysis of colony-forming units (CFU) revealed that collagen-based membranes were more prone to bacterial colonization compared to PTFE membranes. This observation aligns with the surface characteristics of these materials and is well known [15]. Collagen membranes, with their rough texture, provide a more conducive environment for bacterial adhesion, as confirmed by SEM analysis, which showed high bacterial presence on the surfaces of Memlock and Memlock Pliable. On the other hand, PTFE membranes exhibited a smoother surface due to very low porosity, limiting bacterial colonization which is also claimed to be a major advantage by some clinicians and manufactures [16]. The significant difference in bacterial load between collagen-based membranes and PTFE membranes supports the notion that the structural properties of GBR membranes play a critical role in their susceptibility to bacterial contamination. This insight may help also clinicians in selecting the appropriate membrane type based on the clinical scenario, particularly when bacterial infection is a concern. CAP was highly effective in reducing bacterial load across all membrane types. A 5-min CAP treatment already produced a significant reduction in CFU on the Argonaut membrane and extending the treatment to 10 min led to further bacterial reduction, especially on Memlock and Argonaut membranes. Notably, after 10 min of CAP exposure, no viable bacteria were detectable on PTFE membranes, suggesting that CAP is particularly effective during moderate bacterial infections since the synthetic membrane allowed the least colonization. The results from L/D staining and SEM analysis paralleled the findings from CFU quantification, showing a reduction in viable bacteria across all specimens. The time interval of 10 min is also possible to implement in everyday clinical praxis although shorter intervals are desirable. This might be achievable by modifying the intensity and composition of CAP [17].

From a materials science standpoint, plasma treatment is frequently employed to alter the surface properties of polymer films, offering several advantages compared to traditional surface modification methods [18]. According to Azam et al., the presence of functional groups like N–H and C–H can enhance adhesion on dental biomaterials following plasma treatment [19]. In a similar vein, Morent et al. found that plasma treatment introduces C–O, C = O, and O–C = O groups onto polymer surfaces [20]. Recently, Yang et al. suggested that reactive oxygen species may play a role in inhibiting S. mutans growth on zirconia surfaces treated with cold atmospheric plasma [21]. In the current study, it could be speculated that reactive oxygen species could also cause bacterial inhibition, however, the effects of surface carbonyl (C = O) groups on the bacterial proliferation on biomaterials warrants further evaluation.

Although the results of this study are promising, further investigations in vivo are needed. Infections in combination with membranes are usually not only superficial. As of today, anti-infective therapy includes local bactericidal solutions like chlorhexidine to prevent membrane and bone loss. CAP treatment might be superior to the application of bacteria-reducing solutions as Koban and colleagues proved in vitro [22]. Another limit of this study is that the penetration depth of CAP into the membrane was not investigated. It is likely that deeper layers and e.g. bone substitute materials that were covered can be controlled by CAP. Additionally, this study did not investigate the potential effects of CAP on the mechanical properties or biodegradability of the membranes. Since CAP generates reactive species, it is crucial to ensure that its application does not compromise the structural integrity or resorption time of the membranes, especially for bioabsorbable ones. The oral cavity presents a dynamic environment with multiple bacterial species, so future studies should assess the effect of CAP on a broader spectrum of pathogens and in the context of live tissue.

It is obvious that the bacterial colonizations on oral tissues are multi-bacterial and E. faecalis is usually not a dominant species in these infections. However, it is unfortunately not possible to cultivate all specimens of the oral microbiome in-vitro. Moreover, many studies have showed that E. faecalis appears in many oral infections and may vegetate in bone after extraction of an infected tooth and colonize on a dental implant surface, which may cause the failure of the biomaterial [23].

The contamination of a membrane, which leads to an infection could jeopardize the regeneration process and necessitate removal and could be very frustrating from the patient’s perspective. The confirmation of the decontaminative effects of CAP via an in-vivo multi-bacterial colonization model could be beneficial in developing novel strategies in the management of an infection after guided bone/tissue regeneration. However, it should be kept in mind that the CAP treatment should not jeopardize the cytocompatibility.

In conclusion, CAP shows significant promise as an anti-infective treatment for GBR membranes, with the potential to improve outcomes in implant and periodontal surgeries. Future clinical studies will be essential to establish its efficacy and refine its use in routine dental practice.

## Data Availability

No datasets were generated or analysed during the current study.

## Abbreviations

BHI: Brain–Heart-Infusion broth
CAP: Cold atmospheric plasma
CFU: Colony forming units
GBR: Guided bone regeneration
L/D: Live/Dead
PBS: Phosphat buffered saline
PTFE: Polytetrafluoroethylene
SD: Standard deviation
SEM: Scanning electrone microscopy
